# Use of Transpulmonary Pressure Monitoring in the Management of Extrapulmonary Pediatric Acute Respiratory Distress Syndrome With multi organ dysfunction syndrome (MODS): Are We Peepophobic?

**DOI:** 10.1177/1179547619842183

**Published:** 2019-04-12

**Authors:** Mukul Pandey, Dhiren Gupta, Neeraj Gupta, Anil Sachdev

**Affiliations:** Division of Pediatric Intensive Critical Care Unit (PICU), Institute of Child Health, Sir Ganga Ram Hospital, Delhi, India

**Keywords:** Transpulmonary pressure, pediatric acute respiratory distress syndrome, PEEP

## Abstract

Manipulation of positive end-expiratory pressure (PEEP) has been shown to improve the outcome in pediatric acute respiratory distress syndrome (PARDS), but the “ideal” PEEP, in which the compliance and oxygenation are maximized, while overdistension and undesirable hemodynamic effects are minimized, is yet to be determined. Also, for a given level of PEEP, transpulmonary pressure (TPP) may vary unpredictably from patient to patient. Patients with high pleural pressure who are on conventional ventilator settings under inflation may cause hypoxemia. In such patients, raising PEEP to maintain a positive TPP might improve aeration and oxygenation without causing overdistension. We report a case of PARDS, who was managed using real-time esophageal pressure monitoring using the AVEA ventilator and thereby adjusting PEEP to maintain the positive TPP.

## Introduction

Low tidal volumes are clearly useful in patients with pediatric acute respiratory distress syndrome (PARDS), but how to choose a positive end-expiratory pressure (PEEP) is still uncertain. Ideally, mechanical ventilation should provide sufficient transpulmonary pressure (TPP; airway pressure [Paw] minus pleural pressure [Ppl]) to maintain oxygenation while minimizing repeated alveolar collapse or overdistension leading to lung injury. In critical illness, however, there is marked variability among patients in abdominal pressure and Ppl; thus, for a given level of PEEP, TPP may vary unpredictably from patient to patient.^1^ We report a case of PARDS, who was managed using real-time esophageal pressure monitoring using the AVEA ventilator.

## Case Report

**Figure 1.**
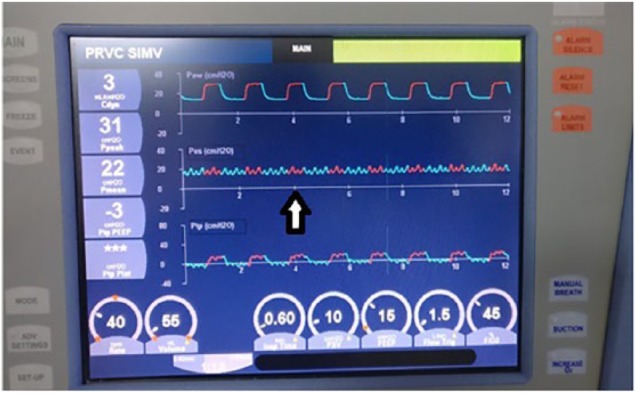
Esophageal pressure of 20 cm of H_2_O (white arrow) was shown.

**Figure 2.**
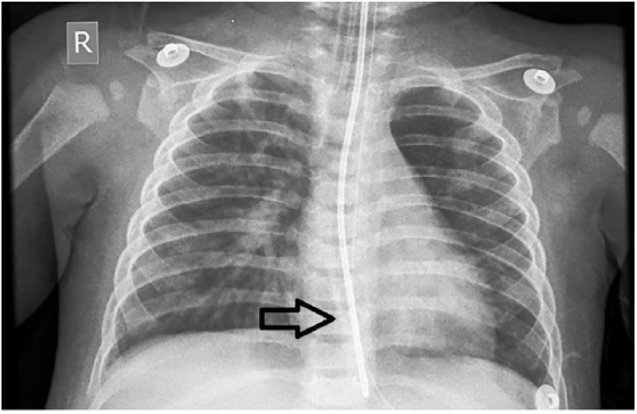
Chest X-ray showing increased chest wall edema leading to poor compliance with the esophageal catheter in situ (black arrow).

A 7-month-old boy (8 kg), immunized for age, was admitted with complaints of fever, cough for 5 days, and fast breathing for 1 day. There was no significant past history and normal birth and developmental history. On examination, vitals were as follows: heart rate: 182/min, blood pressure: 85/55 (65) mm Hg (at 50th centile), temperature: 101.3°F, respiratory rate: 85/min, and oxygen saturation (SpO_2_) (80% on room air and 92% on oxygen by mask at 5 L/min), with anasarca. In the systemic examination, the child was in respiratory failure with crepitation heard over right side of interscapular area. Other systems were normal. Bedside lung ultrasound showed shred sign at the above-mentioned area. Initial diagnosis of bronchopneumonia was made and the child was intubated (4 mm cuffed tube, fixed at 13 cm), started on midazolam, fentanyl infusion, and antibiotics. The child was started on synchronized intermittent mandatory ventilation (SIMV) pressure-regulated volume control (PRVC) mode with settings: tidal volume: 8 mL/kg, PEEP: 8, plateau pressure: 25, rates: 45/min, SpO_2_ 98% on inspiratory oxygen fraction (FiO_2_) 0.4. Positive end-expiratory pressure was chosen as per best compliance and FiO_2_. The clinical condition later deteriorated in the form of desaturation (SpO_2_ 88% on FiO_2_ 0.6), worsening chest wall edema and abdominal distension. Intra-abdominal pressure was increased to 18 cm of H_2_O. To improve oxygenation, the child was ventilated as per the acute respiratory distress syndrome (ARDS) net protocol (low tidal volume and PEEP titration were done by a conventional method from 8 to 15), but there was no improvement in oxygenation. At the PEEP of 15, further titration was abandoned and further adjustment was done by measuring the TPP. The TPP and esophageal pressures were recorded after inserting the esophageal catheter withholding expiration and inspiration for brief periods. By giving a 3-s hold at end-inspiration, transpulmonary plateau (Ptp plat) was recorded, and holding at end-expiration, transpulmonary peep (Ptp peep) was recorded. Esophageal pressure which corresponds to the Ppl was recorded as 20 cm of H_2_O (Figures 1 and 2). To attain Ptp plat between 10 and 15 cm of H_2_O and Ptp peep in the range of 0 to 2 cm of H_2_O, PEEP was increased from 15 (from conventional methods) to 20. Subsequently, the oxygenation improved without hemodynamic instability (Table 1).^1^ Considering worsening renal functions and fluid overload, renal replacement therapy was started in the form of peritoneal dialysis (PD). Positive end-expiratory pressure was also titrated according to the intra-abdominal pressure during PD cycle “in and out” targeting the Ptp plat and Ptp peep. As fluid overload persisted despite PD, continuous renal replacement therapy (CRRT) was done. Slowly, renal functions improved, fluid overload decreased, and the child was extubated on the 7th day. He was discharged 2 weeks after hospitalization and is currently doing well on follow-up.

**Table 1.** Ventilator and physiological parameters at admission, at 24 h (when patient worsened), and at 48 h (after TPP monitoring).

Parameters	At admission	At 24 h	At 48 h
Mode of ventilation	PRVC	PRVC	PRVC
Tidal volume (mL/kg)	8	6	6
PEEP	8	15	20
PaO_2_/FiO_2_ ratio	218	146	311
Plateau pressure	25	30	20
Heart rate (beats/min)	182	170	126
Blood pressure, mm Hg (mean)	85/55 (65)	92/53 (64)	98/55 (67)
Respiratory rate (breaths/min)	45	45	35
TPP at end-inspiratory (Ptp plat) in cm of H_2_O	Not monitored	6	11
TPP at end-expiratory (Ptp peep) in cm of H_2_O	Not monitored	–5	2
Esophageal pressure in cm of H_2_O	Not monitored	20	15
Abdominal pressure (cm of saline)	11	18	9

Abbreviations: PaO_2_/FiO_2_: partial oxygen tension to the inspiratory oxygen fraction; PEEP, positive end-expiratory pressure; PRVC, pressure-regulated volume control; Ptp peep, transpulmonary peep; Ptp plat, transpulmonary plateau; TPP: transpulmonary pressure.

## Discussion

Transpulmonary pressure is the real distending force of the lung parenchyma, and it is calculated as the difference between the Paw and the Ppl.^2^ Increased intra-abdominal pressure and reduced chest wall compliance cause higher Ppl.^3^ Its measurement, therefore, allows partitioning of the lung compliance from the chest wall compliance, which helps to determine what fraction of Paw is applied to overcome lung and chest wall elastance, and the optimal pressure that would prevent the cyclic collapse of alveoli at the end expiration as well as overdistension (shear stress).^4^ Plateau pressure and tidal volume have been shown to be poor surrogates of stress and strain, whereas Ptp has been advocated as a better guide for safe mechanical ventilation.^5^ The only available bedside surrogate of Ppl is esophageal pressure and is based on physiological studies in healthy adults in an upright position.^6^ Esophageal pressure measurement may help in providing optimal TPP while avoiding derecruitment and atelectrauma.

In our case, both intra-abdominal hypertension and decreased chest wall compliance were present. Hence, esophageal pressure–guided ventilation gave us the confidence of increasing the PEEP beyond the conventional levels, and it resulted in the increase in the ratio of partial oxygen tension to the inspiratory oxygen fraction (PaO_2_/FiO_2_) without any hemodynamic instability. Evidence also suggests that the TPP strategy not only improved oxygenation and respiratory system compliance but also prevented extra corporeal membrane oxygenation (ECMO) institution.^1,7^

## Conclusions

Adjusting the ventilation settings targeting the patient’s TPP shows promise for improvement in lung function and survival.^1^ Esophageal pressure monitoring may help to optimize PEEP and driving pressure while avoiding further lung injury in PARDS patients.
